# Editorial: Developmental models 2.0

**DOI:** 10.3389/fcell.2022.1055139

**Published:** 2022-10-12

**Authors:** Alessandra Giorgetti, Ying Gu, Keiichiro Suzuki, Mo Li

**Affiliations:** ^1^ Regenerative Medicine Program, Institut d’Investigació Biomèdica de Bellvitge (IDIBELL), Department of Pathology and Experimental Therapeutics, Faculty of Medicine and Health Sciences, Barcelona University, Barcelona, Spain; ^2^ BGI-Shenzhen, Shenzhen, China; ^3^ Institute for Advanced Co-Creation Studies, Osaka University, Suita, Japan; ^4^ Graduate School of Engineering Science, Osaka University, Toyonaka, Japan; ^5^ Graduate School of Frontier Bioscience, Osaka University, Suita, Japan; ^6^ Bioscience Program, Biological and Environmental Science and Engineering Division (BESE), King Abdullah University of Science and Technology, Thuwal, Saudi Arabia

**Keywords:** pluripotent stem cell, blastoid, organoid, regenerative medicine, 3D cell culture, single-cell “omics”

Human pluripotent stem cells (PSCs), including embryonic stem cells (ESCs) and induced pluripotent stem cells (iPSCs), have enabled the modeling of human development and helped to illuminate mechanisms of monogenic disease, complex disease, and cancer. Lately, the striking ability of PSCs to self-organize has engendered three-dimension (3D) models of human development. The 3D embryonic cell models partially reconstruct the complex architecture of mammalian early embryonic structures and therefore hold great potential for stem cell and developmental studies. In this Research Topic, Gordeeva et al. described a 3D embryoid body differentiation model and compared the spatiotemporal growth and patterning dynamics of embryoid bodies formed from different stem cell origins and culture conditions. Min et al. profiled the proteome and the protein phosphorylation of blastoids–blastocyst-like 3D structures derived from extended pluripotent stem cells (EPSC). By comparing the protein expression profiles of EPSC blastoids with mouse blastocysts, they indicated that glucose metabolism is key to blastoid formation and brought new insights into the similarities and differences between blastoid and blastocyst at the proteome level. In addition, Luijkx et al. provided a comprehensive comparison of current protocols for mouse (Rivron et al., 2018; Kime et al., 2019; Li R et al., 2019; Sozen et al., 2019; Vrij et al., 2019) and human (Fan et al., 2021; Liu et al., 2021; Sozen et al., 2021; Yanagida et al., 2021; Yu et al., 2021; Kagawa et al., 2022) blastoid formation, including the sources of stem cells, the key signaling molecules used in culture medium, and the experimental timelines, and further discussed to which extent these blastoids recapitulate the blastocyst in mouse and human, offering an informative resource to facilitate researchers to study early embryonic developments using blastoids.

Beyond stem cell based early embryonic models, the development of *ex vivo* organoid cultures has gained substantial attention as a model to study regenerative medicine and diseases in several tissues since the seminal work in 2009 (Li M et al., 2019). While human intestinal organoids were among the first human organoid types that were successfully established *in vitro*, many protocols have since been optimized for organoids of many other tissue types, including the pancreas, liver, kidney, stomach, and lung among others. As organoid technology has improved, we have seen a dramatic expansion in its application, providing insights on a range of tissues—both healthy and diseased—as well as in drug development, and organ transplantation. Indeed, organoids can be used to study healthy or diseased tissue and can be generated from embryonic progenitors, adult-derived stem/progenitor cells, tumor samples or differentiated from iPSCs or ESCs. For instance, human intestinal organoids can represent a sufficiently complex *in vitro* model of intestinal tissue from fetal to adult human stages of development, which are otherwise difficult to access. Taelman et al. summarized the differences, advantages, and disadvantages of these intestinal organoid models and discussed the applications of human intestinal organoids. However, current intestinal organoid culture strategies still lack the complex interaction with *in vivo* growth factors, extracellular matrix composition and multi-organ physiology.

Pancreatic organoids were first described in 2013 to model two major devastating diseases: diabetes and pancreatic ductal adenocarcinoma. Casamitjana et al. performed an in-depth review of the characteristics of pancreatic organoids derived from different cell types. They emphasized the potential of pancreatic organoids in future tissue transplantation and personalized medicine and highlighted the limitations of pancreatic organoids to recapitulate tissue differentiation and architecture. Human PSCs offer an unlimited supply of source materials for the generation of 3D human islet-like clusters that are transplantable and ameliorate diabetes in animal models. Yoshihara highlighted the recent progress of generation of stem cell-derived 3D-structured human islets. The author also discussed multiple missing factors in the generation of fully functional human islets, including pancreatic exocrine and immune cell interaction, peripheral neuro-vasculature system, paracrine regulation, organ-organ interaction, and physiological environmental cues.

Chronic kidney disease (CKD) is a general term for heterogeneous disorders affecting kidney structure and function. When kidney function continues to decline, CKD patients may develop end-stage renal disease (ESRD, or kidney failure). Organ transplantation and dialysis continue to represent the only therapeutic options available. However, in the last two decades major advances in stem cell biology, gene editing, and bioengineering are now delivering options in regenerative medicine to treat CKD. Li et al. comprehensively summarized the most prevailing and innovative progress of the current approaches to solving the shortage of donor kidneys and how these approaches can complement each other, with stem cell technologies at the center of these interconnections (Huang et al.). A clear example is the development of stem cell derived kidney progenitors and organoids that may be utilized to provide a reliable source of proximal tubule cells that are needed in commercializing the bioartificial kidney device. Liu et al. reviewed kidney disease model studies using *in vitro* tools including kidney organoids derived from normal human fetal/adult tissues, human PSCs and primary tissues of kidney cancer. They covered many topics including polycystic kidney disease and other genetic kidney diseases, and non-genetic kidney diseases. Both papers also discussed the remaining challenges of translating advances in kidney organoid research into new therapies.

Human cardiac lineages can be differentiated in traditional two-dimensional monolayer culture or by adopting 3D culture methods. Ramirez-Calderon et al. summarized the most advanced 3D methods for deriving human cardiac organoids from human PSCs and discussed the potential applications of cardiac organoids in the pharmaceutical and bioengineering fields, including the emerging question of Severe Acute Respiratory Syndrome CoronaVirus 2 (SARS-CoV-2) infection in the heart.

These primary research and reviews highlight the fact that organoids are heterogeneous in shape and size; moreover, the absence of blood supply and interactions with non-parenchymal cell types limit their potential. For these reasons, future works are needed to standardize organoid cultures, including co-cultures with other cells (mesenchymal, endothelial, neuronal, and immune cells), and to improve cell maturation to generate more faithful models. To this end, improvements in culture methodology could make 3D stem cell models more robust and reproducible. Klein et al. argue that paying due attention to culture environments is critical to improve the reproducibility and translation of preclinical research. They outline the main sources of cell culture environmental instability and deliver best practice recommendations, thus making an important step towards enhancing the physiological relevance of *in vitro* cellular models. Additionally, a higher resolution understanding of the molecular events unfolding during development or tissue homeostasis could guide better 3D differentiation protocols. Single-cell OMICs analysis promise to deliver unprecedented insights into human development and disease pathogenesis. In this Research Topic, Xu et al. applied single-nucleus RNA sequencing (snRNA-seq), which could resolve multiple cell types better than single-cell RNA sequencing (scRNA-seq), to profile cell types, dynamics of cellular composition, and hepatocyte differentiation trajectories during postnatal murine liver development. A complete understanding of the development of complex organs such as the brain requires not only cell type taxonomy (which scRNA-seq excels at) but also spatial information of cells or genes at the organ level. Cheng et al. applied Stereo-seq, a DNA nanoball patterned array-based high-resolution spatial transcriptomic technology, to medial structures in postnatal mouse brain. Their data provided subcellular distribution of 27,330 genes, region-specific gene regulatory networks, 41 cell types localized in different regions. This rich resource for developmental study is accessible as an open and interactive database (https://db.cngb.org/stomics/datasets/STDS0000139?tab=explore). Li et al. performed integrative scRNA-seq and single cell assay for transposase-accessible chromatin sequencing (scATAC-seq) analysis in mesenchymal stem/stromal cells derived from placenta (PMSCs). Their data revealed subsets of PMSCs and nominated potential cis and trans regulatory factors in the subtypes.

Besides high-through profiling of mRNA and chromatin accessibility, mechanistic dissection of regulatory pathways during tissue regeneration pays dividends for improving stem cell differentiation and developing novel therapies. In this regard, Shams et al. showed that signaling through the chemokine receptor CXCR4 is essential to normal early activation, proliferation and self-renewal of muscle stem cells (or satellite cells), which are responsible for regenerating muscle fibers following acute skeletal muscle injury. Among mammals, deer has the unique ability to fully regenerate a lost organ, the antler. Guo et al. explored the gene expression changes during the activation of the pedicle periosteum (harboring stem-like cells) that triggers the initiation of antler regeneration. Their findings suggest that calreticulin (CALR), an androgen response gene, is likely a downstream mediator of androgen hormones for initiating antler regeneration.

The topic editors hope the readers enjoy reading these new papers centered around stem cell models as much as we do. The exciting research advances summarized in this volume will undoubtedly have a positive impact on the translational values of stem cell models.
